# Laryngeal dysfunction is prominent in asthmatic women treated by inhaled corticosteroids

**DOI:** 10.1002/clt2.12211

**Published:** 2022-12-09

**Authors:** Nicolas Migueres, Christina Delmas, Julie Petit Thomas, Hélène Kuntz, Elisabeth Peri‐Fontaa, Philippe Schultz, Michel Velten, Frédéric de Blay

**Affiliations:** ^1^ Division of Asthma and Allergy Department of Chest Diseases Hôpitaux Universitaires de Strasbourg Strasbourg France; ^2^ Department of Pneumology Hôpital Civil de Saverne Saverne France; ^3^ Department of Otolaryngology Head and Neck Surgery Hôpitaux Universitaires de Strasbourg Strasbourg France; ^4^ Department of Epidemiology and Public Health‐EA3430 Faculty of Medicine University of Strasbourg Strasbourg France; ^5^ EA 3072, Fédération de médecine translationnelle Université de Strasbourg Strasbourg France

**Keywords:** asthma, dysphonia, inhaled corticosteroid, phonation disorders

## Abstract

**Background:**

Dysphonia is a frequent comorbidity of asthma and has been suggested to be a local side effect of inhaled corticosteroids due to laryngeal candidiasis. We hypothesized that dysphonia in asthmatics was not due to laryngeal organic lesions but to laryngeal dysfunction during phonation (LDP).

**Objective:**

We compared the frequency of LDP in female asthmatic patients treated with inhaled corticosteroids to female controls.

**Methods:**

We compared 68 asthmatic female patients to 53 female control subjects. Pulmonary function tests were performed and the asthmatic patients classified according to the level of inhaled corticosteroids. Dysphonia was defined as a Vocal Handicap Index ≥18 or GRBAS score ≥2. All patients underwent video laryngo‐strobe examination, analyzed blindly and separately by two otolaryngologists, describing mucosal changes, LDP, or Organic lesions linked to Laryngeal Dysfunction during Phonation (OLDP).

**Results:**

66.2% of the asthmatic patients exhibited dysphonia and 11.3% of controls (*p* < 0.001). No laryngeal candidiasis was found, only 3 patients presented laryngeal mucosa inflammation. LDP was observed in 60.3% of asthmatic patients and 18.9% of controls (*p* < 0.001), and no difference was found for OLDP (11.8% vs. 13.2%). No association was made between LDP, the dosage of inhaled corticosteroid, and bronchial obstruction.

**Conclusions:**

Asthmatic patients were more dysphonic than control subjects. This phenomenon was not explained by mucosal inflammation, laryngeal candidiasis or OLDP. Asthmatic patients had more LDP than controls. There was no relation between LDP, inhaled corticosteroids dosage or bronchial obstruction. These results change our view of inhaled corticosteroid side effects in female asthmatic patients.

## 1 INTRODUCTION

Dysphonia is a disorder characterized by altered vocal quality pitch, loudness, or vocal effort that impairs communication or reduces voice‐related quality of life.^1^ Dysphonia has a lifetime prevalence of 29.9% and a point prevalence of 6.6% in adults aged ≤64 years.^2^ Women are more frequently affected than men, with a female/male ratio of 60:40.^2^, ^3^, ^4^

Dysphonia can be classified into two main categories: organic and functional.^5^ Organic dysphonia are the consequences of aspects not related to the use of voice, with a lot of etiologies.^5^ Functional dysphonia (FD) refers to a voice disturbance that occurs in the absence of structural or neurologic laryngeal pathological characteristics, and may account for 10%–40% of cases referred to multidisciplinary voice clinics.^6^ Muscle tension dysphonia (MTD) is a functional dysphonia, characterized by the presence of Laryngeal Dysfunction during Phonation (LDP), first described by Morrison & Rammage.^5^, ^7^ These dysfunctions have been described as an open posterior commissure (laryngeal isometry), a medio‐lateral constriction during phonation, an antero‐posterior constriction during phonation, and abnormal mucosal waves during phonation.^7^, ^8^, ^9^ Primary MTD involves dysphonia in the absence of current organic vocal fold pathology, whereas secondary MTD indicates dysphonia in the presence of an underlying organic condition.^10^, ^11^ The video‐laryngo‐stroboscopic exam has been described as the gold standard exam for visualizing LDP.^10^, ^12^

Dysphonia has been described as more frequent in asthma than healthy subjects (11.3% vs. 5.5%).^13^ An uncontrolled study on asthma has estimated an association with dysphonia in 50% of cases.^14^ The main hypothesis, supported by only two limited uncontrolled studies,^15^, ^16^ was that dysphonia was caused by inhaled corticosteroids by favoring candidiasis or inducing myopathic bowing of the vocal cords.

Other laryngeal pathologies as Paradoxical Vocal Fold Movement (PVFM) and chronic cough have been associated with asthma.^17^, ^18^, ^19^ The hypothesis of a common mechanism has been proposed for PVFM, chronic cough and MTD, implicating sensory neuronal dysfunction of the larynx.^19^ Indeed, they have overlapping symptomatology and they are associated with both laryngeal dysfunction and upper airway hypersensitivity.^9^, ^20^, ^21^

We hypothesized that the increased of dysphonia in asthmatic patients was due to laryngeal dysfunction during phonation. In order to avoid bias due to sex difference it has been decided to study only women. We compared the frequency of laryngeal dysfunction during phonation between female asthmatic patients treated with inhaled corticosteroids and healthy female controls.

## 2 MATERIALS AND METHODS

### 2.1 Study design

We performed a monocentric comparative study at the Chest Diseases Department of Strasbourg University Hospital from November 2013 to September 2015. Data were collected during two outpatient visits.

The first visit was a respiratory evaluation comprising the following clinical and demographic characteristics: age, height, weight, past medical history (medical and surgical), the presence of rhinitis and familial atopy, inhaled corticosteroid dose, Forced Expiratory Volume in 1 Second (FEV1), and bronchial obstruction (FEV1/Forced Vital Capacity [FVC]). The second visit was a phoniatric evaluation using the Vocal Handicap Index (VHI) questionnaire and GRBAS scale as described below. Inflammatory vocal folds and laryngeal mycosis were reported based on the video‐laryngo‐stroboscopic exam blindly analyzed by two different otolaryngologists (ENTs).

The study was performed in accordance with the Declaration of Helsinki and the International Conference on Harmonization E6 Guideline for Good Clinical Practice, including the agreement of the local ethics committee (CPP Est IV reference:13/16, agreement provided the 03/05/2013; NCT 01999855; ID RCB: 2013‐A00398‐37).

### 2.2 Participants

Two groups of subjects were selected. The asthmatic patient group including 68 asthmatic women aged 18–70 years with persistent asthma (according to GINA 2012 [Global Initiative for Asthma] guideline)^22^ who had been receiving inhaled corticosteroid treatment for at least 3 months. All asthmatic patients were selected in the allergy division in the chest disease department (Strasbourg). They were all outpatients. Asthma diagnosis was based on bronchial reversibility which had to be obtained during the last 2 years before inclusion. If not, a non‐specific hyperresponsiveness to methacholine had to be demonstrated (MEDIPROM FDC 88/Paris/France) and a normal chest X ray. Inhaled corticosteroid (ICS) treatment was considered low if < 500 μg per day, medium if between 500 and 1000 μg per day, and high if > 1000 μg per day (in equivalent dose of beclomethasone). Adherence to inhaled therapy was checked by the clinician. Asthma was assessed as controlled if the patient experienced two time or less symptoms of asthma during day time, without limitation of activities or rescue inhaler use and a normal FEV1.

Exclusion criteria were: upper respiratory tract infections during the past month, endotracheal intubation during the past 3 months, medically confirmed and symptomatic gastroesophageal reflux disease, obesity (BMI >30 kg/m^2^) with abnormal lung function tests, acute sinusitis or chronic rhinitis proven by an ENT with a nasal obstruction proved by mirror test, laryngeal surgery in past medical history, cordal organic lesion not linked to laryngeal dysfunction during phonation (malignant tumor, congenital cysts or mucous cysts, malformations, papillomatosis) at the video‐laryngo‐stroboscopic exam, chronic respiratory failure, heart failure, intermittent asthma, COPD, Karnofski performance status^23^ <80%, pregnancy, or breastfeeding. Smokers with more than 10 pack years or ex‐smokers for more than 5 years with 5 to 10 pack years were also excluded.

The control group included 53 non‐asthmatic female volunteers aged 18–70 years. Recruitment of the controls was performed among voice practitioner students, medical school students, and hospital staff outside the Chest Diseases Department. These controls had no relationship with the department or the ENT division, were subjected to the same exclusion criteria as the patient group in addition to not having asthma.

### 2.3 Paraclinical tests

In the study group, prick tests to common aeroallergens were performed unless they had been performed in the past 5 years (mite, cat, dog, *aspergillus, alternaria*, grass, birch, ash, ragweed, and mugwort pollen; Stallergenes Lab Antony, France). Asthma control assessment was performed according to GINA 2012.^22^

In the control group, only plethysmography was performed. In both groups, lung function was tested using the same plethysmograph (Vmax/VIASYS/Hoechberg/Germany).

### 2.4 Phoniatric tests

Both groups were evaluated by the VHI (French version)^24^ and GRBAS scale.^25^ The VHI is a validated questionnaire measuring voice problems in daily life that consists of 30 items with five response levels scored 0–4. Summarizing the scores results in a total VHI score ranging from 0 to 120. A higher score corresponds to a higher degree of patient‐based vocal handicap. The VHI was normal with an overall score <18. Patients with a score ≥18 were considered dysphonic.

The GRBAS system for describing vocal quality contains five well‐defined parameters: overall grade of hoarseness (G), roughness (R), breathiness (B), asthenic (A), and strained quality (S) of the voice. A 4‐point scale, from 0 to 3, is used for each parameter: “0” equals non‐hoarse or normal, “1” slight, “2” moderate, and “3” severe. Results are denoted as G1RIBIAoS0. Patients with a global rank ≥2 were considered as dysphonic.

Dysphonia was defined by a VHI score ≥18 and/or a GRABS score ≥2.

Phonation quotient, defined by the ratio of vital capacity to maximum phonation time, was used as an indication of air consumption.^26^, ^27^ Phonation quotient has been shown higher in disordered voice.^27^, ^28^ Maximum phonation time was obtained by asking subject to sustain the vowel/a/at a comfortable pitch and intensity level as long as possible following deep inspiration.

For the video‐laryngo‐stroboscopic exam, we used a rigid epipharyngoscop 90° Hopkins model (STORZ/8707 DA/Germany) with ATMOS strobe (21 LED, Camera: ATMOS CAM 31). An audio recording of each voice was obtained with the video strobe recorder. The exam comprised a number of phonatory tasks comprising sustained vowel/a/. The quality of the lining of the vocal folds, the pattern of closure, and the appearance of the free edge were noted. The vocal folds were described in terms of color and the presence of edema, fungal plaques, secretions, or vascular dilatations. The presence of Organic lesions associated with Laryngeal Dysfunction during Phonation (OLDF) (e.g., polyp and nodule) was noted.

The free edge was described as rectilinear or bowed, specifying the location and type of deformation and the presence of a posterior glottis chink was specified. The quality of the swelling and mucosal undulation, as well as the involvement of the ventricular bands were evaluated. The participation of the ventricular bands was listed in four stages: none, light, massive and hard glottal attack. The mucosal undulation was described as ample or reduced, with or without desynchronization. Inflammatory vocal fold lesions and laryngeal mycosis were also reported.

Patients were classified as having Laryngeal Dysfunction During Phonation if they exhibited an anormal vocal fold closure pattern or a participation of ventricular band or an anomaly of mucosal vibration or undulation.

Similarly, video laryngoscopy recordings of patients and control subjects were edited by the first ENT and each recording was identified by the allocated subject number. The recordings were rendered sound free to blind the observers to dysphonic status of each subject.

The video‐laryngo‐stroboscopic results were analyzed blindly and separately by two experienced ENTs specialized in vocal fold pathology. If one of the two ENTs had found LDP associated with a nodule or polyp, the patient was classified as having OLDP. If both specialists found LDP without any organic lesion, the patient was classified as having LDP. If only one of the ENT had found LDP, the subject was not classified as having LDP.

### 2.5 Statistical analysis

Continuous variables were described as means and standard deviation when normaly distributed or as median and interquartile range. Categorical variables were described as the proportion of each modality. The Pearson chi‐squared test or Fisher's exact test were used to compare groups for categorical variables as appropriate. The Student's *t*‐test was used to compare continuous variables between groups. The Mantel‐Haenszel method was used to take into account the effect of age; age was introduced as a four‐level categorical variable defined by the following age classes: 18–30, 30–45, 45–55, 55–70 years.

All statistical tests were two‐sided. Results were considered significant if *p* < 0.05. The statistical analyses were carried out using SAS 9.4 statistical software (SAS Institute).

## 3 RESULTS

### 3.1 Participant characteristics

**FIGURE 1:**
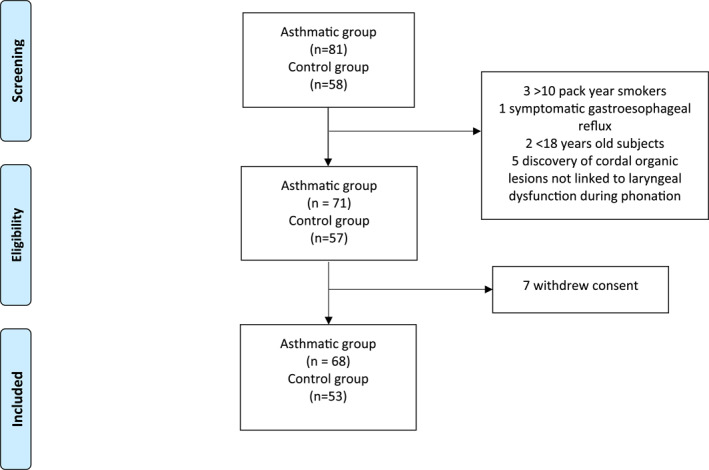
Study inclusion flow chart

121 patients, 68 asthmatics and 53 non asthmatics were included in the study (Figure 1).

As expected, asthmatic subjects were more likely to have rhinitis, polyposis, reflux treatment and had a lower FEV1 and FEV1/FVC (Table 1). Asthmatic patients were also older and had higher BMI, than controls (Table 1).

**TABLE 1:** Population characteristics

	Patients with asthma (*n* = 68)	Control population (*n* = 53)	*p*‐value
Age, years, mean (sd)	46.2 (13.2)	38.1 (13.8)	0.0026
BMI,^a^ kg/m^2^ mean, (sd)	25.1 (4.9)	23.3 (4.0)	0.0157
Obesity *n*, (%)	12 (17.6)	5 (9.4)	0.3048
FEV1, % theoric value, mean (sd)	84.7 (18.4)	99.4 (12.3)	<0.0001
FEV1,^b^ liter, mean (sd)	2.27 (0.63)	2.92 (0.49)	<0.0001
FEV1^b^/FVC,^c^ mean (sd)	72.3 (11.6)	79.8 (7.1)	0.0001
Total lung capacity % theoric value, mean (sd)	103.1 (13.1)	104.8 (11.6)	0.437
Rhinitis *n*, (%)	47 (71.2)	10 (18.9)	<0.0001
Polyposis *n*, (%)	9 (13)	0 (0)	0.0059
Reflux treatment *n*, (%)	16 (23)	0	0.0002
Atopy *n*, (%)	40 (59)	22 (41.5)	
Inhaled corticosteroid dose <500 μg *n*, (%)	10 (14.7)	NA	
Inhaled corticosteroid dose 500–1000 μg *n*, (%)	33 (48.5)	NA	
Inhaled corticosteroid dose >1000 μg *n*, (%)	22 (32.3)	NA	
GINA step 2	6 (9%)	NA	
GINA step 3	25 (37%)	NA	
GINA step 4	30 (44%)	NA	
GINA step 5	4 (6%)	NA	
Asthma control *n*, (%)	7 (10.29)	NA	

^a^ BMI: Body Mass Index.

^b^ FEV1: Forced Expiratory Volume in 1 Second.

^c^ FVC: Forced Vital Capacity. Atopy was defined as having one positive prick test (asthmatic population) or a family history of allergy (control population). Obesity was defined as having a BMI > 30. Data are presented as *n* (%) or average values in the noted units and standard deviation (sd). There were 2 missing values in asthmatic population for rhinitis and 3 for inhaled corticosteroid dosage.

### 3.2 Dysphonia prevalence

Patients with asthma were more likely to be dysphonic than controls whatever the score used: VHI, and/or the GRBAS score (Table 2). They also exhibited higher phonation quotient and lower maximum phonation time. Given that dysphonia increases with age, and that asthmatic subjects were older than controls, we applied the Mantel‐Haenszel method to take into account the effect of age in the comparison between asthmatics and controls. The result was significant (*p* < 0.0001, test for homogeneity: *p* = 0.38), with only a slight reduction (from 5.85 to 5.33) in the magnitude of the relative risk of dysphonia for asthmatics compared to controls.

**TABLE 2:** Dysphonia assessed by VHI,^a^ GRBAS,^b^ combined criteria and phonation quotient

	Patients with asthma (*n* = 68)	Control population (*n* = 53)	Difference [IC 95]	*p*‐value
VHI^a^ ≥ 18 *n*, (%)	38 (55.9)	4 (7.6)	28% [20–36]	<0.0001
GRBAS ≥ 2 *n*, (%)	27 (39.7)	2 (3.8)	20% [12.8–27.1]	<0.0001
VHI^a^ ≥ 18 or GRBAS ≥ 2 *n*, (%)	45 (66.2)	6 (11.3)	32% [23.6–40.3]	<0.0001
Phonation quotient, ml.s^−1^ median IQR	281.5 (182.5, 313.0)	218.3 (174, 240)	63 [22.4–103.9]	0.0016
Maximum phonation time, s median IQR	12 (9, 16.5)	17 (15.21)	5 [2.9–7.0]	<0.0001

^a^ VHI: Voice Handicap Index.

^b^ GRBAS Grade, Roughness Breathiness, Asthenia, Strain Scale. Data are presented as *n* (%) unless otherwise noted.

### 3.3 Video stroboscopic exam

All the video stroboscopic findings are presented in the Online Table S1. No laryngeal candidiasis was reported in asthmatic patients or controls. Inflammation of the laryngeal mucosa was found in only three patients with asthma. Moreover, when considering clinical features during laryngostroboscopy performed by two ENT (see Online Table S1) no major difference was found.

The phoniatric evaluation found a greater prevalence of LDP in the asthmatic population but not of OLDP (Table 3).

**TABLE 3:** Laryngeal dysfunction during phonation and organic lesions linked to laryngeal dysfunction during phonation

	Patients with asthma (*n* = 68)	Control population (*n* = 53)	Difference [IC 95]	*p*‐value
LDP^a^ *n*, (%)	41 (60.3)	10 (18.9)	0.25% [0.18–0.24]	<0.0001
OLDP^b^ *n*, (%)	8 (11.8)	7 (13.2)	1.4% [0–3]	1

^a^ LDP: Laryngeal dysfunction during phonation.

^b^ OLDP: Organic lesions linked to Laryngeal dysfunction during phonation. Data are presented as *n* (%).

### 3.4 Characteristics of patients with dysphonia in the asthmatic group

Asthma characteristics between the groups with and without dysphonia were not different. The asthma control assessed by GINA was not different as well as the FEV1 and FEV1/FVC. Non atopic patients experienced less dysphonia than atopic patients. (78.2% vs. 48.7%, *p* value = 0.02). Obesity, rhinitis, and age were equally present in dysphonic and non dysphonic patients (Table 4). Dysphonic asthmatic patients presented higher median phonation quotient (294 ml.s^−1^ [IQR: 212,350] vs. 210 ml.s^−1^ [IQR: 163,276], *p* = 0.005). and a higher proportion of LDP even though not statistically significant (58% vs. 36%, *p* value = 0.18).

**TABLE 4:** Characteristics of patients with asthma with or without dysphonia

	With dysphonia (VHI ≥18 or GRBAS≥2)	Without dysphonia (VHI <18 and GRBAS<2)	Difference [IC 95]	*p*‐value
	(*n* = 45)	(*n* = 23)		
Age, years, mean (sd)	48.5 (11.99)	41.5 (14.4)	7.0 [−0.5–13.0]	0.06
BMI^a^ ≥ 30 *n*, (%)	7 (15.5)	5 (21.7)	6.2% [0–11]	0.5
Atopy *n*, (%)	19 (48.7)	18 (78.2)	29.5% [18.7–40.3]	0.03
Reflux treatment *n*, (%)	13 (28.9)	3 (13)	15.9% [0–22.4]	0.2
Asthma control *n*, (%)	3 (6.6)	4 (17.4)	1% [−1.3–3.3]	0.2
Rhinitis *n*, (%)	31 (68.9)	16 (69.5)	0.6% [−1.2–2.4]	1.0
FEV1,^b^% theoric value, mean (sd)	84.6 (18.8)	84.9 (17.9)	0.3 [−9.7–9.15]	0.74
FEV1^b^/FVC^c^ mean (sd)	71.9 (10.7)	73.1 (12.7)	1.2 [−7.48–5.01]	0.74
Dry powder inhalation device *n*, (%)	26 (57.7)	13 (56.5)	1.2% [−1.3–3.7]	1.0

^a^ BMI: Body Mass Index.

^b^ FEV1: Forced Expiratory Volume in 1 s.

^c^ FVC: Forced Vital Capacity. Data are presented as *n* (%) unless otherwise noted. There were 6 missing values for atopy and 2 for rhinitis in the group with dysphonia.

### 3.5 Role of inhaled corticosteroids

In the asthmatic population, the prevalence of LDP, and OLDP in relation to the dose of inhaled corticosteroid was not significantly different (Table 5). The prevalence of dysphonia seemed to increase with the dosage of corticosteroid without statistical significance. Phonation quotient was higher in medium and high dosage of inhaled corticosteroid groups.

**TABLE 5:** Prevalence of dysphonia, LDP,^d^ and OLDP^c^ in patients with asthma based on inhaled corticosteroid dose

	Inhaled corticosteroid dose <500 μg (*n* = 10)	Inhaled corticosteroid dose 500–1000 μg (*n* = 33)	Inhaled corticosteroid dose >1000 μg (*n* = 22)	*p*‐value
VHI^a^ ≥ 18 or GRBAS ≥ 2 *n*, (%)	3 (30)	23 (69.7)	16 (72.7)	0.054
OLDP^c^ *n*, (%)	0	3 (9.1)	5 (22.7)	0.186
LDP^d^ *n*, (%)	7 (70)	20 (60.6)	12 (54.5)	0.74
FEV1^b^/FVC,^e^ mean (sd)	76.3 (8.4)	72.5 (12.1)	71.5 (11.6)	0.6
Phonation quotient, ml.s^−1^ median IQR	177.0 (153.8205.2)	238.0 (177.0,346.0)	290.0 (260.0,309.0)	0.009

^a^ VHI: Voice Handicap Index.

^b^ FEV1: Force Expiratory Volume in 1 s.

^c^ OLDP: Organic Laryngeal Dysfunction during Phonation.

^d^ LDP: Laryngeal Dysfunction during Phonation.

^e^ FVC: Forced Vital Capacity. Data are presented as *n* (%) unless otherwise noted. There were 3 missing values for inhaled corticosteroid dosage.

No OLDP were found in the low ICS group. LDP seemed to be more prevalent in the low ICS group than in the medium ICS group or in high ICS group, but the difference between ICS groups was not significant (*p* = 0.74).

## 4 DISCUSSION

We confirmed that patients with asthma are more likely to be dysphonic than healthy controls. Among the asthmatic patients, we observed more LDP.

To carefully define dysphonia, we used composite criteria with two validated tests. The VHI has demonstrated strong internal consistency, reliability, and test‐retest stability.^29^, ^30^, ^31^

The perceptual evaluation of voice by an ENT is a good complement to the VHI, as the GRBAS scale is examiner‐based.^32^ In contrast, other studies^2^, ^14^ have used non‐validated questionnaires or only one question to assess the prevalence of dysphonia in asthmatic patients and the general population. The differences in dysphonia prevalence in the literature could be explained by the absence of standardized questionnaires to define dysphonia. Our result is also confirmed by the difference of phonation quotient between asthmatic and control group which has been shown to be higher in disordered voice.^28^

Dysphonia prevalence was lower when we used the GRBAS scale. This suggests that the handicap induced by dysphonia is not always quantified by perceptual analysis of the voice. In contrast, some patients with a GRBAS ≥2 exhibited a VHI under 18 and did not complained of voice problems.

Video laryngo‐stroboscopy features were described blindly by two different ENT specialized in phoniatric diseases and allowed detailed physical examination of the vibratory margin of the vocal fold and was helpful for determining the presence or not of organic lesions. Spiegel et al. reported that stroboscopic exam modified the diagnosis in 47% of cases.^33^ The method has been used to elaborate the classification of MTD.^34^ For these reasons, the lack of stroboscopic exam is a limitation in studies on dysphonia in asthmatic patients.^16^, ^35^

Multiple etiologies have been proposed to explain dysphonia in patients with asthma. Williams et al.^16^ performed laryngeal examinations in 14 asthmatic patients using inhaled corticosteroid with persistent dysphonia. Only 3 patients had laryngeal candidiasis and 9 (64%) had bilateral adductor vocal cord deformity with bowing of the cords on phonation. The hypothesis was steroid‐induced myopathy. These findings were not confirmed^36^ by a dysphonia diary, indirect laryngoscopy, and aerodynamic measures in 72 dysphonic asthmatic patients taking inhaled corticosteroid (Nebuhaler® or Turbuhaler®). Only 2 (2%) were found to have vocal cord bowing, and no laryngeal candidiasis was reported. In both studies,^16^, ^36^ no stroboscopy was performed and laryngeal examination was performed by only one examiner. In another study, a total of 22 patients complaining of dysphonia were examined by two ENTs using video laryngo‐ stroboscopy^37^; 58% had mucosal changes, 43% had apposition abnormalities, and 40% had supraglottic hyperfunction, suggesting multi‐factorial etiology. No control group was assessed.

Importantly, we found that patients with asthma were more dysphonic than control subject and did not have more Organic lesions linked to Laryngeal Dysfunction during Phonation (OLDP). Laryngeal mucosal inflammation was reported in only three asthmatic patients, and no laryngeal candidiasis was found. Our results were close to those found by Park et al.,^13^ who performed a large cross‐sectional study comparing subjective voice complaints and organic laryngeal diseases (vocal cord polyps, vocal cord nodules, or laryngitis).

In our study, patients with asthma demonstrated more LDP than control. LDP was higher in dysphonic asthmatic patients than non dysphonic. However, the difference was not significant. Larger studies specifically designed for these objective appear to be needed to confirm these first results.

Non atopic asthmatic patients experienced dysphonia more. This finding could be in accordance with other results showing that capsaicin evoked cough responses were more important in non‐atopic asthmatic.^38^ Indeed Vertigan et al. showed that laryngeal dysfunction is common in the cough hypersensitivity syndrome and hypothesized that it may contribute to its mechanisms.^9^

Phonation quotient was increased in patients treated by medium and high dosage inhaled corticosteroids, dysphonia rate seemed to increase too. As all our asthmatic subjects were taking inhaled corticosteroid, it was not possible to determine whether these results were associated with anti‐asthmatic treatment or with asthma.

However, we did not objectively measure a dose‐effect relationship between LDP, inhaled corticosteroids dosage and bronchial obstruction (FEV1/FVC).

As demonstrated by this study MTD is prevalent in patients with asthma. Voice therapy, in association with patient education and vocal hygiene, has been described efficient in primary MTD.^10^, ^39^, ^40^ More robust evidence of efficiency was found for primary MTD than dysphonia with vocal nodule, mass lesions or with unilateral vocal fold paralysis.^41^

The principal strengths of this study are a rigorous definition of dysphonia with 2 validated tests and stroboscopic analysis performed blindly by 2 otolaryngolists. The limitations are: Asthmatic population was significantly older than the control population, and dysphonia increases with age.^42^ However, we showed that the bias resulting from this discrepancy was very limited, and we were able to estimate an adjusted relative risk that was significantly greater than 1. Asthmatic population exhibited higher BMI than control and dysphonia has been described more prevalent in obese population.^43^ Although, in our study, we did not observed a significantly higher rate of patients with a BMI >30. The video‐laryngo‐stroboscopic exam was realized during phonatory tasks and not connected speech. Also, eight patients with asthma used reflux treatment without medically proven reflux. These patients could have developed dysphonia secondary to undiagnosed reflux. If so, they were treated and were not symptomatic.

## 5 CONCLUSION

Dysphonia was more frequent in asthmatic women treated by inhaled corticosteroid than a control female population and this phenomenon was not explained by mucosal inflammation, laryngeal candidiasis or OLDP. Patients with asthma had also more LDP than controls. There was no relation between LDP and inhaled corticosteroids dosage nor bronchial obstruction. These results change our views of the side effects of inhaled corticosteroid in female asthmatic patients and the treatment of dysphonia in asthma.

## AUTHOR CONTRIBUTIONS

**Nicolas Migueres:** Conceptualization (Equal); Formal analysis (Equal); Methodology (Equal); Validation (Equal); Writing – original draft (Lead); Writing – review & editing (Lead). **Christina Delmas:** Conceptualization (Equal); Investigation (Equal); Validation (Equal). **Julie Petit Thomas:** Conceptualization (Equal); Investigation (Equal); Methodology (Equal); Validation (Equal). **Hélène Kuntz:** Conceptualization (Equal); Investigation (Equal); Validation (Equal). **Elisabeth Peri‐Fontaa:** Conceptualization (Equal); Investigation (Equal); Methodology (Equal); Supervision (Equal); Validation (Equal). **Philippe Schultz:** Conceptualization (Equal); Data curation (Equal); Investigation (Equal); Supervision (Equal); Validation (Equal). **Michel Velten:** Conceptualization (Equal); Formal analysis (Lead); Methodology (Lead); Supervision (Equal); Validation (Equal). **Frédéric de Blay:** Conceptualization (Equal); Funding acquisition (Equal); Investigation (Equal); Methodology (Equal); Validation (Equal); Writing – original draft (Equal); Writing – review & editing (Equal).

## CONFLICTS OF INTEREST

This study was partly funded by ADIRAL (Association d’Aide aux Insuffisants Respiratoire d’ALsace).

## Supporting information

Supplementary Material 1

## Data Availability

The data that support the findings of this study are available on request from the corresponding author.
