# Supplementary material for: Laryngeal dysfunction is prominent in asthmatic women treated by inhaled corticosteroids
Source: Clin Transl Allergy. 2022 Dec 9;12(12):e12211. doi: 10.1002/clt2.12211 (PMC9734276; doi:10.1002/clt2.12211)
Supplement: Supplementary file 1 — Supplementary Material 1 [file CLT2-12-e12211-s001.docx]

**Online table: Laryngostroboscopic constatation**

|  | First ENT | | Second ENT | |
| --- | --- | --- | --- | --- |
|  | **Control (n=53)** | **Patients with asthma (n=68)** | **Control (n=53)** | **Patients with asthma (n=68)** |
| Pharyngeal mucous membrane | 53 ( 100%) normal aspect  0 swelling aspect  0 inflammatory aspect  0 mycosis aspect  0 secretion aspect | 64 ( 94.1%) normal aspect  0 swelling aspect  4 (5.9%) inflammatory aspect  0 mycosis aspect  0 secretion aspect | 52 (98%) normal aspect  0 swelling aspect  0 inflammatory aspect  0 mycosis aspect  1 (2%) secretion aspect | 66 (97%) normal aspect  0 swelling aspect  0 inflammatory aspect  0 mycosis aspect  2 (3%) patients with secretion |
| Vocal fold mucous membrane | 52 (98%) normal aspect  1 (2%) swelling aspect  0 inflammatory aspect  0 mycosis aspect  0 secretions aspect | 57 (84%) normal aspect  2 (3%) swelling aspect  3 (4.5%) inflammatory aspect  0 mycosis aspect  6 (8.5%) secretion aspect | 43 (81%) with normal aspect  0 swelling aspect  1(1.8%) inflammatory aspect  0 mycosis aspect  9 (16.8%) patients with secretions | 45 (66.1%)with normal aspect  0 swelling aspect  3 (4.5%) inflammatory aspect  0 mycosis aspect  20 (29.4%) patients with secretion |
| Free edge aspect and deformation | 48 (90.5%) rectilinear free edge  5 (9.5%) nodul deformation  0 pseudonodul deformation  2 (3%) unilateral deformations  3 (4.5%) bilateral deformations | 51 (75%) rectilinear free edge  11 (16%) nodul deformation  6 (9%) posterior pseudo nodul deformation  8 (12%) unilateral deformation  9 (13%) bilateral deformation | 51 (96%) rectilinear free edge  2 (4%) nodul deformation  0 pseudonodul deformation  2 (4%) bilateral deformation | 66 (97%) rectilinear free edge  2 (3%) nodul deformation  0 pseudonodul deformation  1 (1.5%) unilatéral deformation  1 (1.5%) bilateral deformation |
| Vocal fold closure | 31 (58.5%) complete closure  14 (26.5%) posterior glottis chink  3 (5.6%) longitudinal glottis chink  0 oval shaped glottis chink  5(9.4%) hourglass shaped glottis chink | 26 (38%) complete closure  22 (32%) posterior glottis chink  8 (12%) longitudinal glottis chink  2 (3%) oval shaped glottis chink  10 (15%) hourglass shaped glottis chink | 37 (70%) complete closure  11 (20.7%) posterior glottis chink  1 (1.8%) longitudinal glottis chink  0 oval shaped glottis chink  4 (7.5%) hourglass shaped glottis chink | 43 (63.2%) complete closure  15 (22%) posterior glottis chink  3 (4.5%) longitudinal glottis chink  4 (5.8%) oval shaped glottis chink  3 (4.5%) hourglass shaped glottis chink |
| Ventricular band participation | 26 (49%) none  22 (41.5%) mild  5 (9.5%) permanent  0 hard glottal attack | 8 (11.7%) none  18 (26.5%) mild  39 (57.3%) permanent  3 (4.5%) hard glottal attack | 43 (81.1%) none  6 (11.4%)mild  4 (7.5%) permanent  0 hard glottal attack | 31 (45.5%) none  9 (13.2%) mild  25 (36.8%) permanent  3 (4.5%) hard glottal attack |
| Mucosal vibration/ synchronization | 49 (92.4%) ample  4 (7.5%) reduced  3 (5.6%) desynchronization | 34 (50%) ample  34 (50%) reduced  7 (10.2%) desynchronization | 45 (85%) ample  8 (15%) reduced  0 desynchronization | 41(60.2%) ample  27 (39.8%) reduced  1(1.5%) desynchronization |
| Mucosal undulation | 49 (92.4%) ample  4 (7.6%) reduced  0 desynchronization | 33 (48.5%) ample  35 (51.5%) reduced  2 (3%) desynchronization | 51 (96.2%) ample  2 (3.8%) reduced  0 desynchronization | 52 (76.4%) ample  16 (23.5%) reduced  0 desynchronization |
